# Achieving Complete Response in Metastatic Prostate Cancer With Triplet Therapy and Local Radiation: A Case Report

**DOI:** 10.7759/cureus.84310

**Published:** 2025-05-17

**Authors:** Katsuki Muramoto, Fumihiko Urabe, Keigo Sakanaka, Hajime Onuma, Soshi Kadena, Yuma Goto, Mana Nakata, Tatsuya Shimomura, Takahiro Kimura

**Affiliations:** 1 Department of Urology, Jikei University School of Medicine, Tokyo, JPN

**Keywords:** androgen receptor signaling inhibitors, metastatic castration sensitive prostate cancer, prostate cancer (pca), prostate cancer radiation therapy, triplet therapy

## Abstract

Triplet therapy, consisting of androgen deprivation therapy, docetaxel, and androgen receptor signaling inhibitors, has been approved for the treatment of metastatic castration-sensitive prostate cancer (mCSPC) and is primarily recommended for high-volume disease. A 68-year-old man presented with suspected prostate cancer following the detection of lumbar spine metastases. Prostate-specific antigen (PSA) was as high as 69.76 ng/mL at the first visit. Imaging revealed widespread metastases in the bones and lungs, and a biopsy confirmed prostate cancer with a Gleason score of 4+5=9 (cT3b, N1, M1c). Triplet therapy (surgical castration, docetaxel, and darolutamide) was initiated, resulting in a rapid decline in PSA to <0.01 ng/mL within four months. Metastatic lesions progressively regressed, and a complete response was achieved on imaging 17 months after treatment initiation. However, repeat prostate biopsy revealed residual viable tumor cells (Gleason score of 4+5=9), prompting local radiation therapy to the prostate. Two months after radiation, the patient remained on darolutamide monotherapy, with PSA persistently <0.01 ng/mL.

This case highlights the potential for achieving a complete response in mCSPC with triplet therapy. Even in metastatic disease, first-line treatment may lead to a complete response, underscoring the need for further studies to identify patients who may benefit most from this approach.

## Introduction

The treatment landscape for metastatic castration-sensitive prostate cancer (mCSPC) has evolved significantly in recent years [1]. While androgen deprivation therapy (ADT) alone was previously the standard of care, the addition of docetaxel or androgen receptor signaling inhibitors (ARSIs) to ADT has now become the preferred treatment approach [2-4]. Furthermore, two recent randomized trials have demonstrated that triplet therapy, comprising ADT, docetaxel, and an ARSI, offers superior efficacy compared to the combination of ADT and docetaxel alone [5,6]. Based on the findings of the ARASENS study, darolutamide was approved for the treatment of mCSPC in Japan in January 2023. Nevertheless, the majority of prostate cancer deaths are metastatic, and complete response (CR) is rarely achieved [3]. Radiation therapy is not usually used for prostate cancer with high-volume metastases, but it is one of the first-line treatments for localized prostate cancer. Here, we report a case of a patient who achieved a complete response following triplet therapy (surgical castration, docetaxel, and darolutamide) in combination with radiation therapy for mCSPC.

## Case presentation

**Figure 1:**
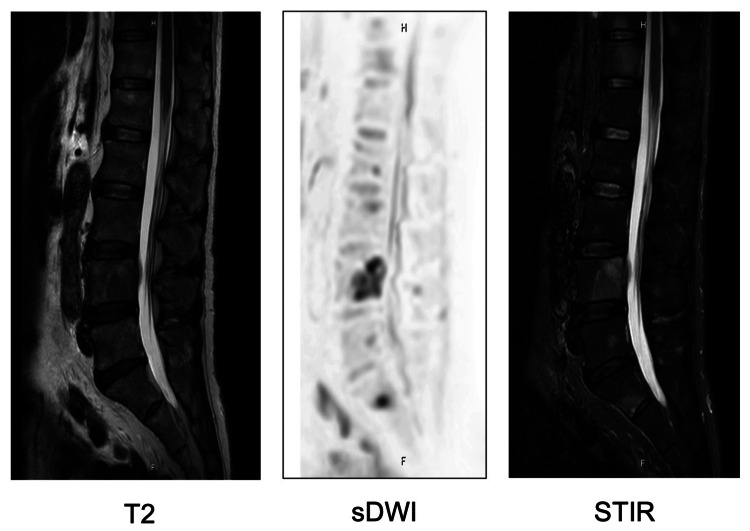
MRI demonstrating osseous metastases in the lumbar spine prior to treatment initiation. sDWI: standard diffusion-weighted imaging; STIR: short tau inversion recovery

**Figure 2:**
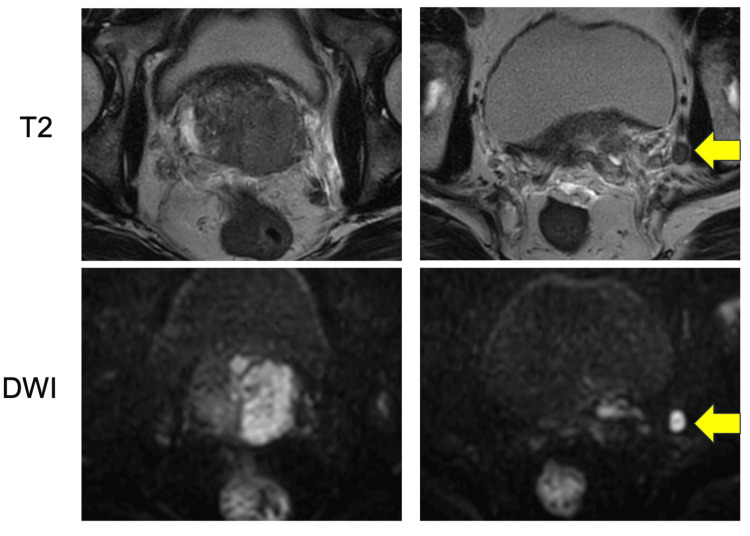
MRI revealing prostate cancer and left pelvic lymph node involvement before treatment initiation. The arrows show enlarged left pelvic lymph node. DWI: diffusion-weighted imaging

A 68-year-old man presented to the hospital with low back pain and was subsequently referred to our institution after lumbar spine magnetic resonance imaging (MRI) revealed an osseous lesion (Figure 1). At the time of the initial visit, there were no symptoms other than back pain, including lower urinary tract symptoms. His initial prostate-specific antigen (PSA) level was markedly elevated at 69.76 ng/mL, and prostate MRI suggested prostate cancer with left seminal vesicle invasion (cT3b) and left pelvic lymph node metastasis (Figure 2).

**Figure 3:**
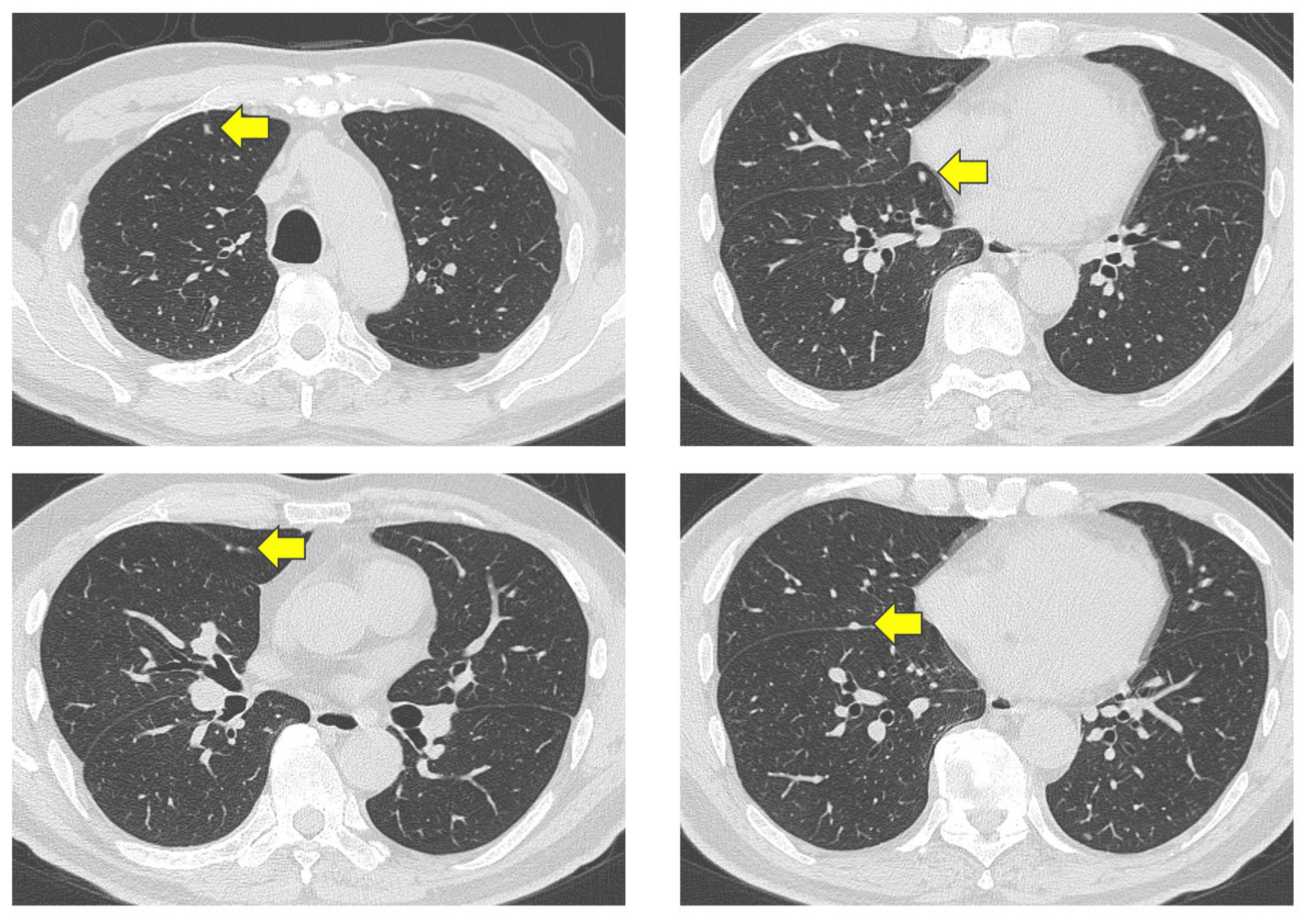
CT scan showing multiple metastatic lesions in the right lung before treatment initiation. Small nodules were scattered in the right lung and showed disappearance in the same area (arrows).

**Figure 4:**
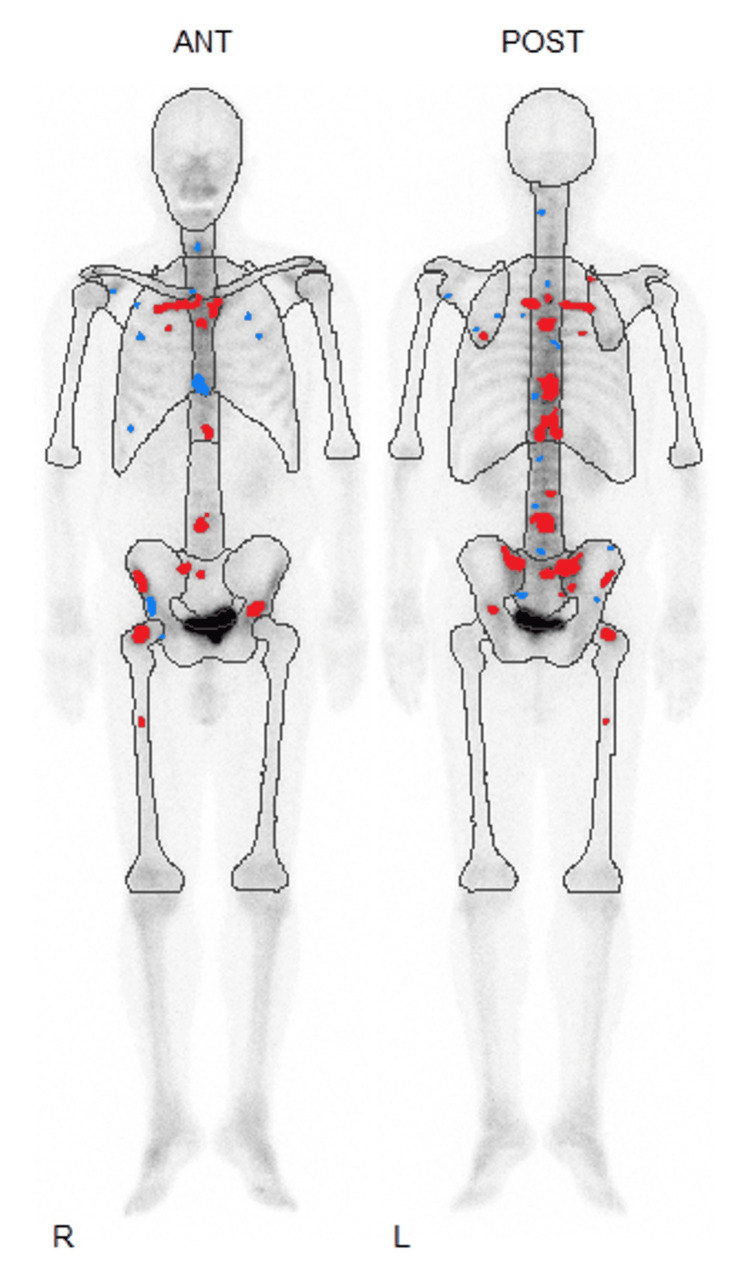
Bone scintigraphy showing systemic bone metastases before treatment initiation. Analysis of hot spots in bone scintigraphy shows areas of high (red dots) and low (blue dots) potential for abnormal accumulation. ANT: anterior; POST: posterior

A prostate needle biopsy confirmed the diagnosis of prostate cancer with a Gleason score of 4+5=9 (positive cores: 3/6). Computed tomography (CT) imaging revealed right lung metastasis (Figure 3), and bone scintigraphy revealed systemic bone metastases, establishing a clinical stage of cT3bN1M1c (Figure 4).

**Figure 5:**
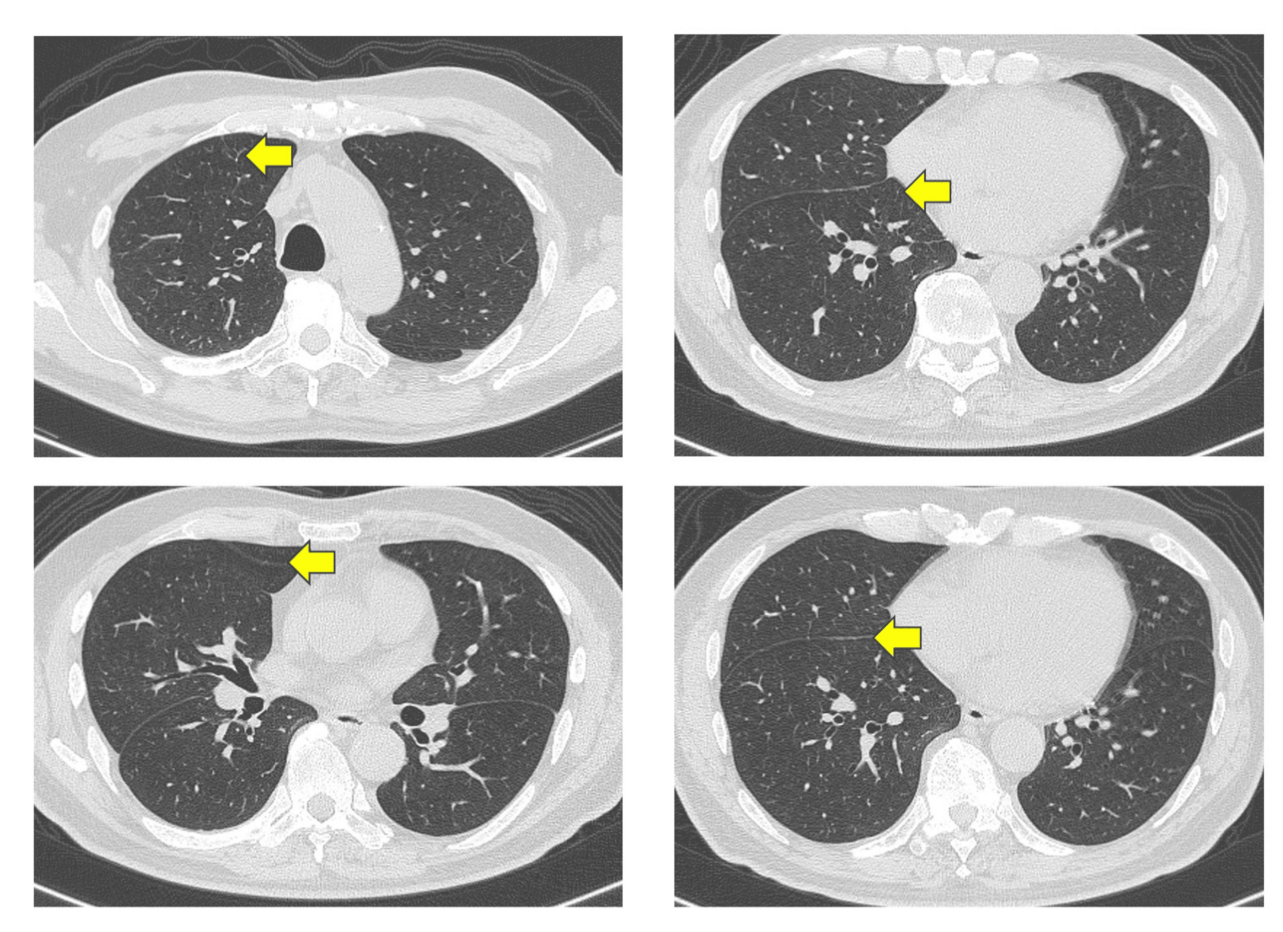
CT scan demonstrating the resolution of lung metastases following treatment. Small nodules were scattered in the right lung and showed disappearance in the same area (arrows).

Neuron-specific enolase (NSE) was normal, but alkaline phosphatase (ALP) (193 U/L) and lactate dehydrogenase (LDH) (246 U/L) were elevated. The patient was initiated on triplet therapy comprising ADT (castration), docetaxel (75 mg/m^2^ every three weeks), and darolutamide (1,200 mg/day, administered twice daily). After completing three cycles of docetaxel, imaging showed the resolution of lung and lymph node metastases (Figure 5).

**Figure 6:**
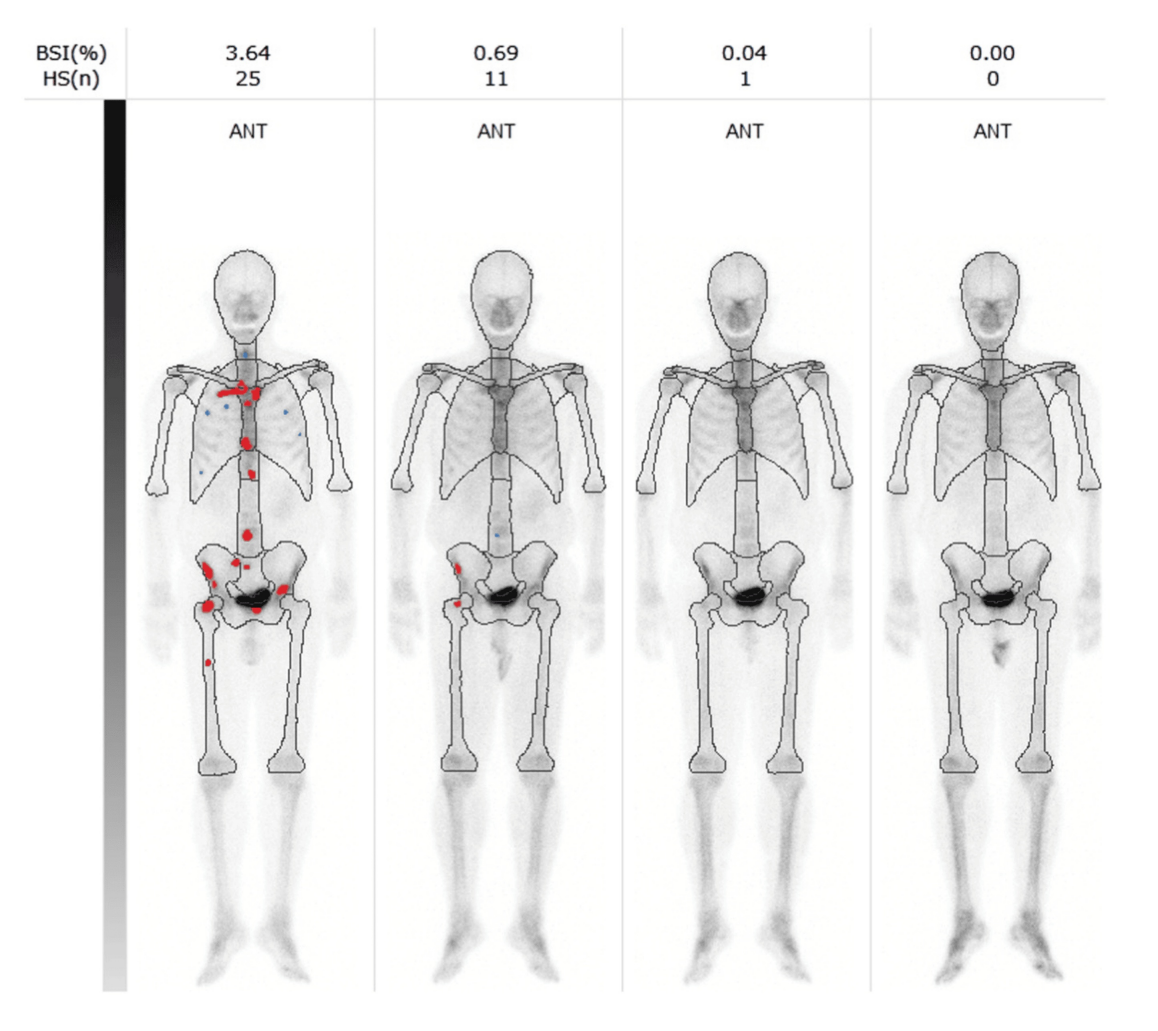
Serial bone scintigraphy images illustrating changes in bone metastases following treatment. Red dots indicate areas of likely abnormal accumulation in the bone scintigraphy. Light gray indicates normal bone overlap, while dark gray indicates normal excretion of nuclides, such as in the bladder. BSI: bone scan index; HS: hotspot; ANT: anterior

**Figure 7:**
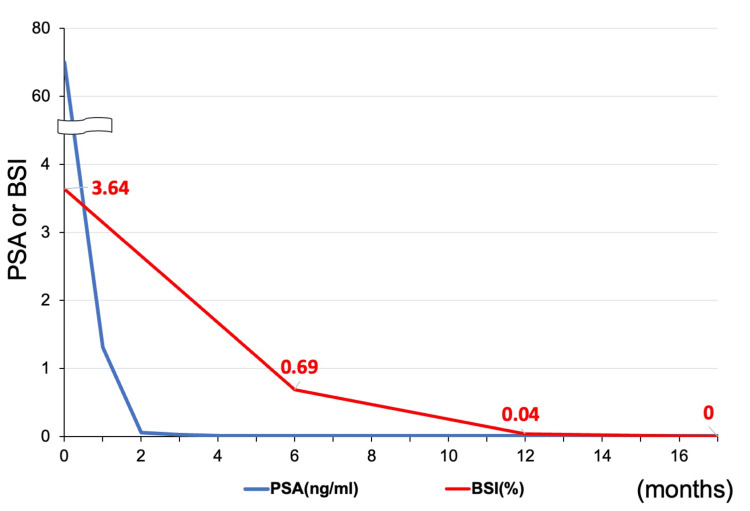
Graph depicting changes in PSA levels (ng/mL) and bone scan index (BSI, %) over the course of treatment. PSA: prostate-specific antigen; BSI: bone scan index

However, during the fourth cycle, the patient developed oral mucositis (grade 1), a skin rash (grade 1), and bilateral leg edema (grade 3), necessitating the discontinuation of docetaxel. Darolutamide was continued at the same dosage following docetaxel discontinuation. PSA levels declined rapidly, reaching <0.01 ng/mL within four months of initiating triplet therapy. Additionally, the bone scan index, calculated from bone scintigraphy, progressively decreased to 0.00% within 17 months (Figures 6, 7). ALP and LDH levels also decreased quickly from the start of treatment.

**Figure 8:**
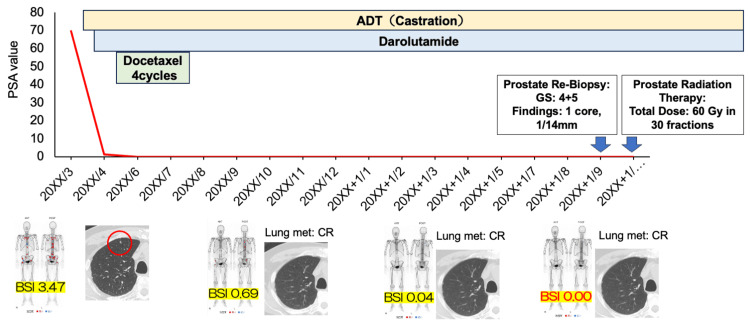
Timeline of treatment and results. The red circle indicates one lung metastasis. The red line indicates changes in PSA levels over the course of treatment. BSI: bone scan index; PSA: prostate-specific antigen; CR: complete response

A follow-up prostate MRI, performed after the resolution of all metastatic lesions, showed no evidence of residual cancer, indicating a radiographic complete response (CR). Despite the absence of detectable disease on imaging, a repeat prostate biopsy was performed, which revealed residual prostate cancer with a Gleason score of 4+5=9 (positive core: 1/12), confirming the presence of viable tumor cells. Consequently, the patient underwent prostate-directed radiation therapy 18 months after the initiation of systemic treatment (total radiation dose: 60 Gy/30 fr, as per the patient’s request for treatment completion within six weeks) (Figure 8). At the latest follow-up, five months have elapsed since the completion of radiation therapy. The patient remained on darolutamide monotherapy, and PSA levels continued to be undetectable at <0.01 ng/mL.

## Discussion

Reports of CR achieved with triplet therapy in mCSPC patients are extremely rare. The ARASENS study compared ADT plus docetaxel plus darolutamide with ADT plus docetaxel plus placebo in patients with mCSPC. The results demonstrated a significant improvement in overall survival and a prolonged time to progression to castration-resistant prostate cancer in the triplet therapy group [6]. Additionally, subgroup analysis indicated a superior PSA response in the triplet therapy cohort. In the overall study population, the proportion of patients achieving undetectable PSA levels (<0.2 ng/mL) at any point before the data cutoff (October 25, 2021) was more than twice as high in the triplet therapy group (67%) compared to the placebo group (29%) [7].

Recently, our group reported real-world data on triplet therapy (ADT+docetaxel+darolutamide), showing a 99% reduction in PSA in 82.2% of the patients and an imaging response in 97.8% [8]. Similarly, in the present case, lung metastases disappeared within three months of initiating treatment, PSA reached <0.01 ng/mL within four months, and bone metastases gradually resolved, with all metastatic lesions disappearing by 17 months.

A key aspect of this case was the decision to perform a prostate re-biopsy after the resolution of metastases. Although imaging showed no residual evidence of prostate cancer, the biopsy confirmed the presence of viable tumor cells in the prostate. This finding highlighted the importance of histopathological assessment, as imaging alone may not always be sufficient to confirm a CR. Ultimately, CR was achieved through the addition of prostate-directed radiation therapy as a local treatment.

The increasing availability of prostate-specific membrane antigen positron emission tomography has been reported to offer superior detection of both primary and metastatic prostate cancer lesions compared to conventional CT and bone scintigraphy [9,10]. However, in Japan, it remains unapproved for insurance coverage, limiting its accessibility. Additionally, false-negative results have been reported in cases of low PSA levels or neuroendocrine differentiation [9]. Therefore, despite being a conventional method, prostate re-biopsy remains a valuable diagnostic tool in selected cases.

Currently, the National Comprehensive Cancer Network guidelines recommend triplet therapy as a category 1 treatment for high-volume mCSPC, while for low-volume disease, it remains a category 2B recommendation. A network meta-analysis demonstrated that triplet therapy significantly improves overall survival compared to ARSI-based doublet therapy; however, this survival benefit has not been observed in patients with low-volume disease [11]. While this suggests that triplet therapy is primarily beneficial for high-volume mCSPC, achieving CR may justify its use in select low-volume cases.

Given that 10-20% of prostate cancers may be undetectable on MRI, re-biopsy should be considered in cases that exhibit CR on imaging [12]. If residual disease is confirmed, additional radiotherapy may be warranted. Although robust evidence is lacking, long-term PSA suppression may provide justification for the discontinuation of darolutamide in select cases. Furthermore, if limited metastatic recurrence occurs, multidisciplinary team management should be considered.

At present, no established criteria exist to predict which patients are most likely to achieve imaging-defined CR following triplet therapy. As a result, further clinical data are needed to refine patient selection and optimize treatment strategies.

## Conclusions

In conclusion, this study presents a unique case of mCSPC in which triplet therapy led to CR. This case highlighted that, even in mCSPC, CR can be achieved with first-line therapy. Further studies are warranted to identify patient subgroups who may derive the greatest benefit from triplet therapy.
